# Exosomal and non-exosomal miRNA expression levels in patients with HCV-related cirrhosis and liver cancer

**DOI:** 10.18632/oncotarget.28036

**Published:** 2021-08-17

**Authors:** Alisa A. Petkevich, Aleksandr A. Abramov, Vadim I. Pospelov, Natalya A. Malinina, Elena I. Kuhareva, Natalya V. Mazurchik, Olga I. Tarasova

**Affiliations:** ^1^Genetic Research Laboratory of Advanced Therapy Department, Peoples’ Friendship University of Russia (RUDN University), Moscow, Russian Federation; ^2^Advanced Therapy Department, Peoples’ Friendship University of Russia (RUDN University), Moscow, Russian Federation

**Keywords:** microvesicles, miRNA, cirrhosis, liver cancer, saliva

## Abstract

Patients with HCV-related cirrhosis are at risk for liver cancer development. For these patients miRNAs may serve as preclinical markers, which expression levels are deregulated in cancer and which are stable to the damaging factors partly through complex formation with proteins or packaging into exosomes. In this research we have tried to identify what miRNA fraction in plasma – exosomal or not packed into exosomes (non-exosomal) – is stronger associated with primary liver cancer. The second question was whether saliva miRNA expression levels – both exosomal and non-exosomal – are associated with primary liver cancer. We evaluated exosomal and non-exosomal miRNAs – let-7a-5p, -16-5p, -18a-5p, -21-5p, -22-3p, -34a-5p, -103a-3p, -122-5p, -221-3p, -222-3p – in plasma and saliva of patients with HCV-related liver cirrhosis (*n* = 24), primary liver cancer (*n* = 24) and healthy volunteers (*n* = 21). Relative expression level was calculated with normalization of exosomal miRNA to exosomal miRNA-16-5p, non-exosomal miRNA to non-exosomal miRNA-16-5p and as a ratio of exosomal miRNA to non-exosomal miRNA. In this study, non-exosomal miRNAs (let-7a, miRNA-21-5p, -22-3p, -103a, -122-5p, -221-3p and 222-3p) normalized to non-exosomal miRNA-16-5p showed strong association with liver cancer in plasma. Three miRNAs, those with the mostly pronounced change of expression levels in plasma, – miRNA-21-5p, 122-5p, 221-3p – were detected in saliva. In contrast, exosomal miRNAs show stronger association with primary liver over non-exosomal miRNAs when working with saliva. Thus, depending on the examined biological material both miRNA fractions may serve as a valuable source for diagnostic and prognostic data.

## INTRODUCTION

Liver cancer despite its relatively low incidence rate with a geographically varied global burden is a fatal disease due to its high mortality rate: 5-year survival rate changes from 2% to 33% depending on the stage of the disease at the time of the diagnosis [1, 2]. Hepatocellular carcinoma (HCC) is the most common type of primary liver cancer reaching 80–90% of all primary liver cancers [3]. In 20% of all cases, this is an out of the blue disease developing without any liver cirrhosis [4]. Nevertheless, liver cancer may develop due to a wide variety of reasons, like hepatitis B virus, hepatitis C virus (HCV) related liver cirrhosis, dietary exposure to aflatoxin B1 etc., [5].

Amongst those who are infected with chronic HCV, 15–30% would progress to cirrhosis and 1–5% are expected to die due to decompensated cirrhosis and liver cancer [6]. Anyway, despite the relative low incidence rate of malignancy of an HCV-related cirrhosis and the era of direct antivirals, there is an accumulated pool of patients with HCV-related liver cirrhosis who are at risk group of liver cancer development. Surely, in these cases liver cancer is not that much an out of the blue disease but anyway it may be clinically silent during early stages of the disease, thus, methods for identifying molecular changes developing in pre-clinical stages of the disease are essential.

A valuable source of such molecular information is microvesicles including exosomes, serving as an intracellular whatsapp with huge buffer-exchange volumes. Exosomes are indentified as microvesicles with a size range 30–150 nm [7] or 40–160 nm [8] or 100–200 nm [9], in this paper we assume that exosomes are 30–200 nm microvesicles. The microvesicles may contain different proteins, DNA and various RNA including miRNA, a small non-coding nucleic acid of 18-24 nucleotides involved in epigenetic regulation of the gene expression [10]. Exosomes along with other microvesicles may serve as a target delivery system and mechanism for preserving the structure of the molecules being their defense from different enzymes [11]. Not packed into exosomes miRNAs (hereinafter non-exosomal miRNAs) some years ago were supposed to be non-stable molecules vulnerable to blood RNAses, last years, they are shown to be detectable in the biological fluids in protein and lipoprotein complexes, that prevents their degradation.

The first question to answer in this study was to determine which fraction of extracellular miRNAs in plasma – packed into exosomes (hereinafter, exosomal) or not packed into exosomes (hereinafter, non-exosomal) – is stronger associated with primary liver cancer in plasma. The second question was whether saliva miRNAs – both exosomal and non-exosomal – are associated with primary liver cancer.

## RESULTS

### Exosome determination in the samples

Exosomes in the samples were identified with indirect method of photon cross-correlation spectroscopy: peaks were identified in a range from 50 nm to 250 nm in all 8 exosomal samples (4 from blood plasma and 4 from saliva) in the control and every study group indicating that in these samples there are microvesicles of exosomal size range, which is 50–200 nm. In all 8 exosome-depleted samples (4 from blood plasma and 4 from saliva) in the control and every study group there were peaks in a range from 50 nm to 70 nm also indicating the presence of the microvesicles of exosomal size range. This may be due to incomplete elimination of microvesicles from plasma and saliva samples. Approximate number of CD63 positive microvesicles in a sample was determined with CD63 based ELISA analysis (shown in Table 1). This data allows to assume the amount of exosomes in exosome-depleted samples is below the meaning of the least amount of the exosomes, provided by manufacturer in calibration curve, and this value can be neglected in further work.

**Table 1:** Approximate numbers of CD63 positive microvesicles detected with CD63 based ELISA

	Study group 1^1^		Study group 2^2^		Control group^3^	
	plasma	saliva	plasma	saliva	plasma	saliva
Exosome depleted samples	not detected^4^	not detected	not detected	not detected	not detected	not detected
Exosomal samples	~2,6 E+10	~0,9 E+10	2,5 E+10	~1,1 E+10	2,5 E+10	~0,8 E+10

^1^Study group 1 – patients with cirrhosis, *n* = 24, ^2^Study group 2 – patients with liver cancer, *n* = 24, ^3^Control group – healthy volunteers, *n **=*** 21, ^4^The least amount of the exosomes, provided by manufacturer in calibration curve, is 0,07 × 10^10^, sensitivity of the test is 1 μg protein equivalent detection.

### miRNA concentration in samples

MiRNA concentration in all samples was determined, in exosomal samples concentrations varied from 0,89 μg/mcl to 4,8 μg/mcl; in exosome depleted samples from 2,77 μg/mcl to 7,5 μg/mcl.

### Expression level of exosomal and non-exosomal miRNAs

**Figure 1:**
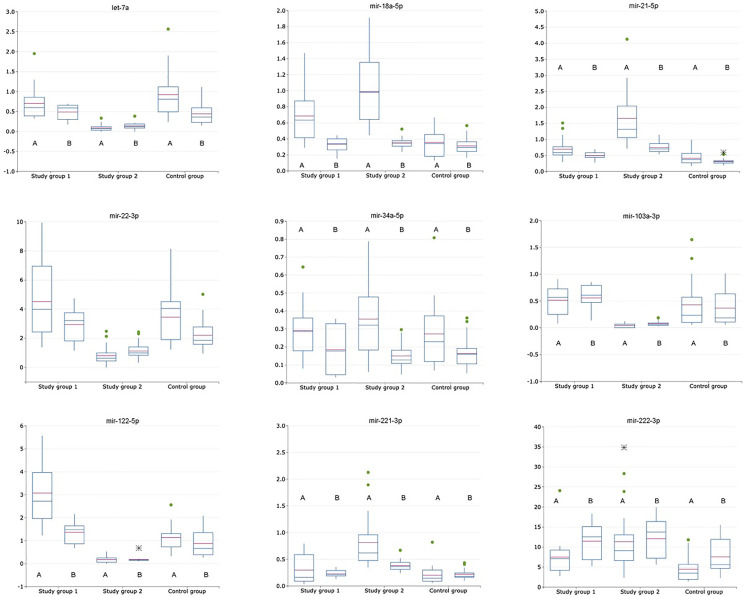
Relative expression of exosomal and non-exosomal miRNAs (normalized to the corresponding miRNA-16-5p) in plasma of patients with HCV-related cirrhosis (study group 1, *n* = 24), liver cancer (study group 2, *n* = 24) and healthy volunteers (control group, *n* = 21); A is for exosomal miRNA; B is for non-exosomal miRNA.

**Figure 2:**
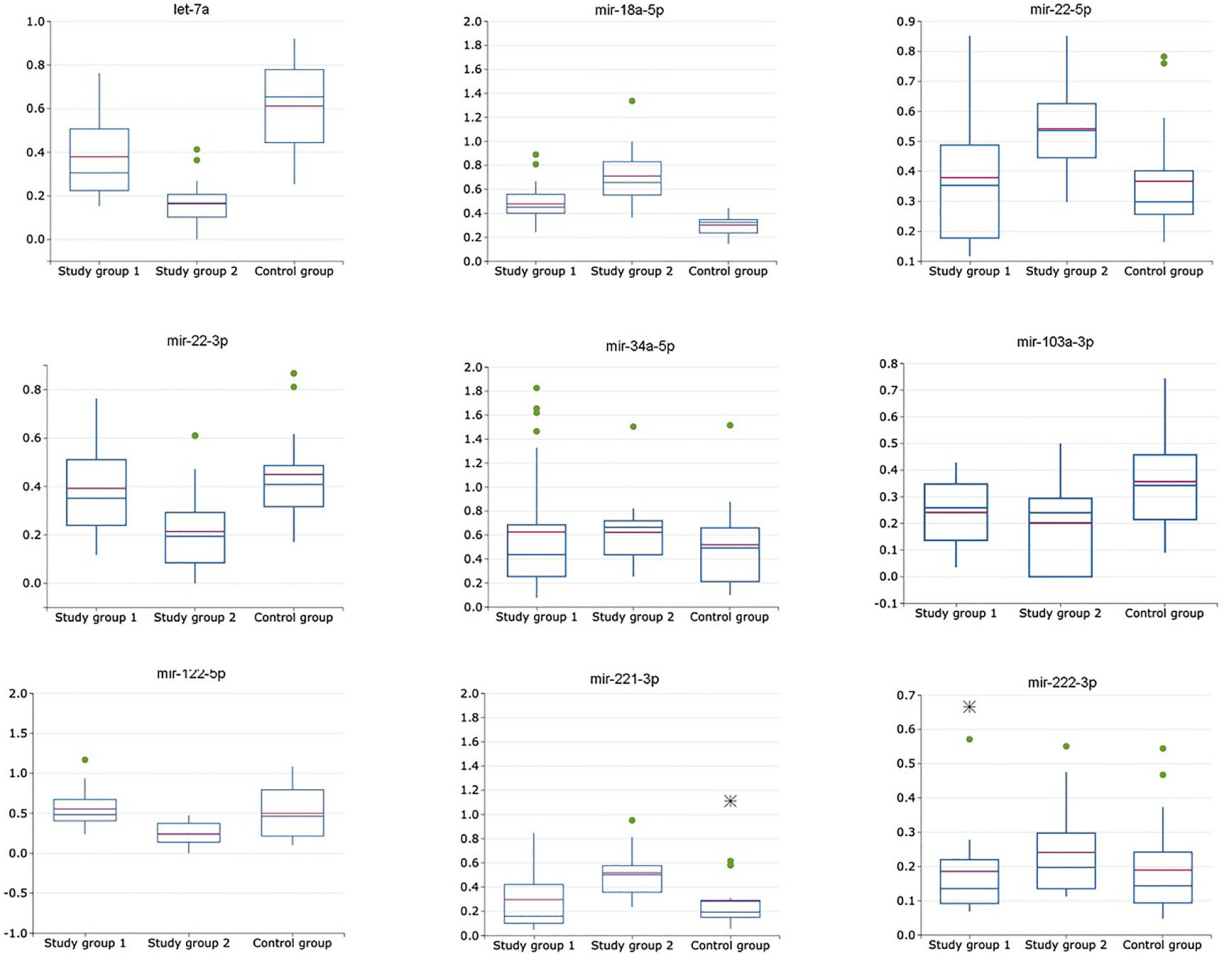
Relative expression of miRNAs (exosomal to non-exosomal miRNAs ratio) in plasma of patients with HCV-related cirrhosis (study group 1, *n* = 24), liver cancer (study group 2, *n* = 24) and healthy volunteers (control group, *n* = 21).

**Figure 3:**
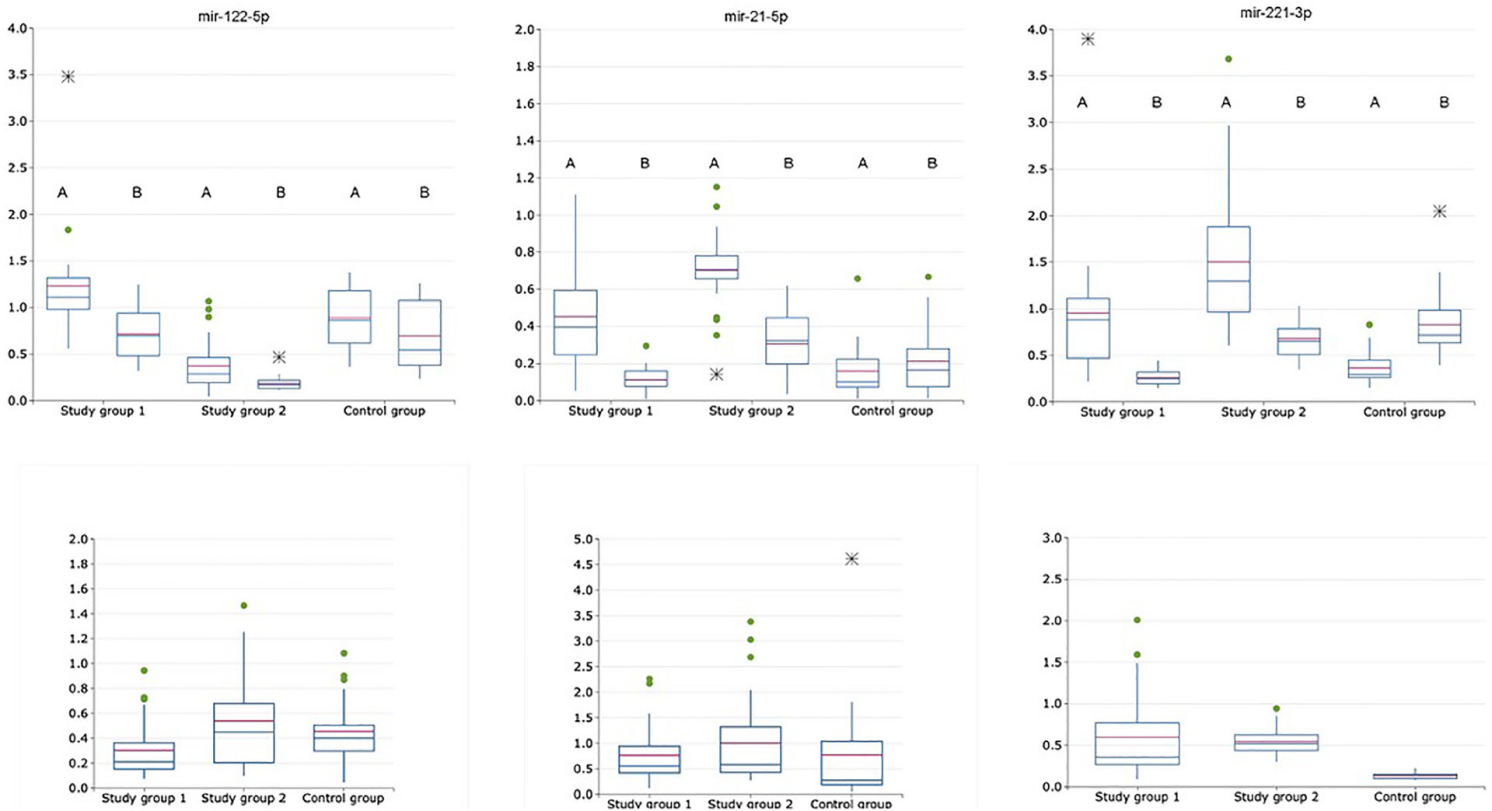
Relative expression of exosomal and non-exosomal miRNAs normalized to the corresponding miRNA-16-5p (upper raw) and normalized as exosomal to non-exosomal miRNAs ratio (lower raw) in saliva of patients with HCV-related cirrhosis (study group 1, *n* = 24), liver cancer (study group 2, *n* = 24) and healthy volunteers (control group, *n* = 21); A is for exosomal samples; B is for non-exosomal samples.

From the set of examined miRNAs two miRNAs – miRNA-145-5p and miRNA-224-5p – were eliminated because there were not detected (*C*_t_ value of > 35) both in plasma exosomal and exosome-depleted samples in control and every study group. Expression levels of the following miRNAs – let-7a-5p, miR-16-5p, miR-18a-5p, miR-21-5p, miR-22-3p, miR-34a-5p, miR-103a-3p, miR-122-5p, miR-221-3p, miR-222-3p – were determined both in plasma exosomal and exosome-depleted samples in control and every study group (Table 2) and three miRNAs (miRNA-122-5p, -21-5p, -221-3p) were identified in saliva (Table 3). Expression level of each of these miRNAs was calculated as following: exosomal miRNA-X/exosomal miRNA-16-5p (for plasma miRNAs Figure 1, for saliva miRNAs Figure 3), non-exosomal miRNA-X/non-exosomal miRNA-16-5p (for plasma miRNAs Figure 1, for saliva miRNAs Figure 3), exosomal miRNA-X/non-exosomal miRNA-X (for plasma miRNAs Figure 2, for saliva miRNAs Figure 3), where miRNA-X is a target/studied miRNA.

**Table 2:** Significance of exosomal and non-exosomal miRNA expression level difference in plasma in healthy volunteers, hepatocellular carcinoma and liver cirrhosis

Study groups	N	let-7a	mir-18	mir-21	mir-22	mir-34a	mir-103a	mir-122	mir-221	mir-222
HCC/LC	e/n-e	0.00001	0.00011	0.00181	0.00103	0.49113	0.15647	5.34649E-7	0.00077	0.09031
HCC/HV	e/n-e	1.41859E-12	9.78214E-10	0.00028	0.00005	0.12003	0.00117	0.00053	0.00042	0.10107
LC/HV	e/n-e	0.00016	0.00002	0.42080	0.17264	0.21668	0.00688	0.24411	0.42716	0.46360
HCC/LC	e/miR16	1.75056E-9	0.00336	1.05143E-6	2.32496E-9	0.12160	2.52250E-10	4.3856e-14	0.00002	0.02189
HCC/HV	e/miR16	5.28507E-9	3.93833E-8	1.19869E-8	1.34904E-8	0.08330	0.00005	5.44032E-11	8.88518E-7	0.00031
LC/HV	e/miR16	0.07108	0.00010	0.00035	0.05313	0.34435	0.22388	9.72938E-8	0.09019	0.00536
HCC/LC	n-e/miR16	6.61017E-11	0.22048	5.58919E-8	4.50614E-9	0.12806	8.2614e-13	1.8268e-17	5.67705E-8	0.32600
HCC/HV	n-e/miR16	2.10138E-6	0.08421	3.230255e-14	0.00003	0.29906	0.00002	3.67392E-7	2.04322E-7	0.00078
LC/HV	n-e/miR16	0.28479	0.22776	7.54786E-7	0.01288	0.25809	0.00002	0.00123	0.26207	0.00209

N for normalization method. HCC for a group of patients with hepatocellular carcinoma. LC for a group of patients with liver cirrhosis. HV for a group of patients with healthy volunteers. e/n-e for exosomal miRNA to non-exosomal miRNA ratio. e/miR16 for exosomal miRNA to exosomal miRNA-16 ratio. n-e/miR16 for non-exosomal miRNA to non-exosomal miRNA-16 ratio.

**Table 3:** Significance of exosomal and non-exosomal miRNA expression level difference in saliva in healthy volunteers, hepatocellular carcinoma and liver cirrhosis

Study groups	N	mir-122	mir-21	mir-221
HCC/LC	e/n-e	0.00712	0.13659	0.31759
HCC/HV	e/n-e	0.20546	0.19050	1.3845e-14
LC/HV	e/n-e	0.02305	0.47498	0.00008
HCC/LC	e/miR16	8.52313E-9	0.00073	0.00814
HCC/HV	e/miR16	5.18581E-7	1.15359E-12	3.37936E-8
LC/HV	e/miR16	0.00754	0.00007	0.00040
HCC/LC	n-e/miR16	8.522020274789183e-12	4.10744E-6	2.3056e-13
HCC/HV	n-e/miR16	2.04210E-8	0.04090	0.04874
LC/HV	n-e/miR16	0.41124	0.00916	5.20617E-9

N for normalization method. HCC for a group of patients with hepatocellular carcinoma. LC for a group of patients with liver cirrhosis. HV for a group of patients with healthy volunteers. e/n-e for exosomal miRNA to non-exosomal miRNA ratio. e/miR16 for exosomal miRNA to exosomal miRNA-16 ratio. n-e/miR16 for non-exosomal miRNA to non-exosomal miRNA-16 ratio.

MiRNAs with the most changed relative expression levels (calculated as normalized to the corresponding miRNA-16-5p) compare to control in plasma samples from the cancer study group were following miRNAs: miRNA-21-5p (exosomal increased in 4,04 ± 0,54; non-exosomal increased in 2,35 ± 0,32), miRNA-122-5p (exosomal decreased in 0,15 ± 0,013; non-exosomal decreased in 0,13 ± 0,02), miRNA-221-3p (exosomal increased in 4,01 ± 0,34; non-exosomal increased in 1,77 ± 0,21).

### Non-exosomal miRNA relative expression levels calculated as a ratio of target non-exosomal miRNA to non-exosomal miRNA-16-5p ratio

Expression levels of non-exosomal miRNA-34a-5p and miRNA-18a-5p normalized to non-exosomal miRNA-16-5p did not significantly differ (*p* > 0,05) amongst both study and control groups and were eliminated from the following analysis (Table 2). Expression levels of miRNA-let7a and miRNA-221-3p did not significantly differ (*p* > 0,05) between the cirrhotic study group and the control group, allowing to suppose there is no significance influence of cirrhosis development on plasma expression levels of these miRNAs. MiRNA-222-3p expression level did not significantly differ (*p* > 0,05) between the cirrhotic and the cancer study group. ROC analysis for miRNA set consisting of let-7a, miRNA-21-5p, -22-3p, -103a, -122-5p, -221-3p and 222-3p was performed with the values, obtained from the cancer study group and the control group, following AUC values were obtained – 0,98 (sensitivity – 0,9; specificity – 0,98) (Figure 1). Also, ROC analysis for this miRNA set was performed with the values, obtained from the cancer study group and the cirrhotic study group, following AUC values were obtained – 0,97 (sensitivity – 0,9; specificity – 0,99). Non-exosomal miRNA relative expression levels calculated as a ratio of target non-exosomal miRNA to non-exosomal miRNA-16-5p ratio are shown in Figure 1.

### Exosomal miRNA relative expression levels calculated as a ratio of target exosomal miRNA to exosomal miRNA-16-5p ratio

Expression level of exosomal miRNA-34a-5p did not significantly differ (*p* > 0,05) amongst both study and control group and was eliminated from following analysis. Expression levels of exosomal let-7a, miRNA-22-3p, -103a-3p and -221-3p did not significantly differ (*p* > 0,05) between the liver cirrhosis group and control group. (Table 2) ROC analysis for miRNA set consisting of let-7a, miRNA-18a-5p, miRNA-21-5p, -22-3p, -103a, -122-5p, -221-3p and 222-3p was performed with the values, obtained from the cancer study group and the control group, following AUC values were obtained – 0,96 (sensitivity – 0,82; specificity – 0,95) (Figure 1). Also, ROC analysis for this miRNA set was performed with the values, obtained from the cancer study group and the cirrhotic group, following AUC values were obtained – 0,86 (sensitivity – 0,8; specificity – 0,78). Generally, expression levels of exosomal miRNAs were significantly (*p* > 0,05) decreased in comparison with non-exosomal miRNA expression levels in all study groups. Exosomal miRNA relative expression levels calculated as a ratio of target exosomal miRNA to exosomal miRNA-16-5p ratio are shown in Figure 1.

### Relative miRNA expression levels calculated as exosomal miRNA to non-exosomal miRNA expression levels ratio

This approach to relative miRNA expression level calculation resulted in elimination of miRNA-34a-5p and miRNA-222-3p from the following analysis due to no statistically significant difference in obtained numbers (*p* > 0,05) (Table 2). Change in expression levels of miRNA-21-5p, -22-3p, -122-5p and -221-3p calculated with this method were not significantly different (*p* > 0,05) between the liver cirrhosis group and the control group. ROC analysis for miRNA set consisting of let-7a, miRNA-18a-5p, miRNA-21-5p, -22-3p, -103a, -122-5p and -221-3p was performed with the values, obtained from the cancer study group and the control group, following AUC values were obtained – 0,78 (sensitivity – 0,61; specificity – 1,0). Also, ROC analysis for this miRNA set was performed with the values, obtained from the cancer study group and the cirrhotic group, following AUC values were obtained – 0,69 (sensitivity – 0,47; specificity – 0,91). Relative miRNA expression levels calculated as exosomal miRNA to non-exosomal miRNA expression levels ratio are shown in Figure 2.

### Exosomal and non-exosomal miRNA expression levels in saliva

Three miRNAs with the most pronounced changes in expression levels in plasma for both exosomal and exosome-depleted samples (normalized to the corresponding miRNA-16-5p) were miRNA-21-5p, miRNA-122-5p and miRNA-221-3p (Table 3). These miRNAs were determined in exosomal and exosome-depleted samples obtained from saliva. Calculating exosomal miRNA to non-exosomal miRNA ratio resulted in no significant difference (*p* > 0,05) for these miRNAs except for miRNA-221-3p between the cancer study group and control group. Thus, the following analysis was performed with exosomal and non-exosomal miRNA expression levels normalized to the corresponding miRNA-16-5p. ROC analysis for exosomal miRNA set consisting of these three miRNAs was performed with the values, obtained from the cancer study group and the control group, following AUC values were obtained – 0,88 (sensitivity – 0,73; specificity – 1,0). Also, ROC analysis for exosomal miRNA set was performed with the values, obtained from the cancer study group and the cirrhotic group, following AUC values were obtained – 0,54 (sensitivity – 0,78; specificity – 0,5). For non-exosomal miRNAs there were the following values: 0,77 (sensitivity – 0,66; specificity – 0,78) for cancer study group vs control; 0,56 (sensitivity – 0,62; specificity – 0,6) for the cirrhotic study group vs cancer study group. Thus, either exosomal and non-exosomal miRNAs-21-5p, -122-5p and -221-3p determined in saliva do not allow to differ cancer and cirrhotic cases. Exosomal and non-exosomal miRNA expression levels in saliva are shown in Figure 3.

## DISCUSSION

MiRNAs are putative molecular harbingers of liver cancer development, which are quite stable molecules detectable in both blood plasma and saliva. Whereas mRNA, rRNA, and tRNA are degraded within several seconds after being placed in nuclease rich extracellular environment [12], most circulating in biological fluids miRNAs are resistant to RNAse activity, pH change and in some researches are supposed to be resistant to multiple numbers of freeze-thaw cycles [13] This resistance is explained by association of miRNAs with protein or lipoprotein complexes or packaging into exosomes what indicates that miRNAs are not just byproducts of cellular activity but are the means of intentional intercellular connections.

In this research, we investigated non-exosomal and exosomal miRNAs expression levels normalized to the corresponding miRNA-16-5p and exosomal miRNA and non-exosomal miRNA ratio in the groups of patients with cirrhosis and liver cancer and in healthy volunteers in blood plasma and the most promising miRNAs in saliva. Expression levels of exosomal miRNAs were significantly (*p* > 0,05) decreased in comparison with non-exosomal miRNA expression levels in our study for all detected miRNAs in all groups. These data are supported by other studies: in 2011 Turchinovich A, et al. showed, that the amount of plasma supernatant miRNA was ~80 times higher compared to the plasma pellet, thus indicating the overwhelming majority of circulated miRNAs are supernatant (non-exosomal) and is bound to Ago2 protein, but it is worth to admit that exosomes in this paper were defined as vesicles of 50–100 nm [11] Arryo J. D. et al. provided convincing data that about 90% of circulating miRNAs molecules are non-exosomal and associated with different proteins protecting them from external damaging factors [14].

Taking into account a huge role of the exosomes in carcinogenesis and a problem of miRNA normalization it seems to us reasonably despite a minority of exosomal miRNA fraction not to ignore this part of investigated miRNAs. Thus, in this research we used exosomal miRNA X to non-exosomal miRNA X ratio as one of the possible ways of miRNA normalization, where X is the target miRNA. We hypothesized that evaluation of exosomal miRNAs would be more precise compare to non-exosomal miRNAs, but in our research this assumption was not proved: sensitivity and specificity of exosomal miRNAs based panel are significantly inferior to non-exosomal miRNA based panel both for the control group and the study group with liver cancer patients (0, 82 and 0,95 vs 0,9 and 0,98) and for the study groups with patients with HCV-related cirrhosis and liver cancer (0,8 and 0,78 vs 0,9 and 0,98). These results may allow to assume non-exosomal miRNAs have more significant prognostic value compare to exosomal miRNAs. However, it should be admitted, that the sensitivity and specificity were calculated for the whole set of miRNAs with statistically significant difference of relative expression levels between the two groups (cancer and control; cancer and cirrhosis). Such a high values of sensitivity and specificity of non-exosomal miRNAs panel may be explained by the particularities of the sample: all the patients with primary liver cancer involved in this study were at severe stages of the disease (II and more according to AJCC system, 2018), and cancer cells are supposed to be one of the main players in the miRNA expression dysregulation [15]. Nevertheless, there should be admitted that the prevailing part of exosomes circulated in blood plasma are produced by platelets, and their role in primary liver cancer are not that evident and clear, so there is a risk for identification the changes in miRNAs expression levels that are not the consequences of the target process [16].

Generally, identification of non-exosomal miRNAs seems to us more inconvenient than identification of exosomal miRNAs, as far as for the latter case there is a wide variety of commercial kits, some of them are precipitation based and are easy to carry out, allowing relatively standardized exosome isolation method. As for non-exosomal miRNAs there should be performed elimination of microvesicles from the biological fluid, which should allow following investigation of biological fluid for non-exosomal miRNAs. One of the most obvious option is multistep ultracentrifugation which is not a convenient technique for the implementation into clinical routine practice.

The miRNAs for the investigation were chosen according to their target genes, as one of the possible reasons of mRNA profile changes taking place in cancer and adjacent tissues may be the miRNA circulating in blood plasma both in exosomes and in the protein or lipoprotein complexes, which are released by cancer cells and are even able to act like hormones [17]. The second condition was sharing common target genes for several miRNAs. The third condition was identification of the miRNAs by other research groups in the biological fluids and cases of interest for us. Thus, according to MirAnalyze database, let-7a-5p, miRNA-16-5p, -21-5p share common target genes as follows: B-cell translocation gene 2 (*BTG 2*), and fibroblast growth factor receptor substrate 2 (*FGFR2*), which change their expression in cancer [18–20]. MiRNA-21-5p was one of the most significantly changed in cancer, what may allow to make a rough assumption that the expression level of its target genes should be decreased. This may find a support in published papers: expression level of *BTG2* was significantly reduced in HCC tissues (*p* = 0.05, *n* = 44) [28]. The other research demonstrates similar results: *BTG2* expression was significantly suppressed in human HCC compared to adjacent non-cancerous tissues. (*n* = 77) [19]. Nevertheless, the expression level of *FGFR2*, which is also a target gene for miRNA-21-5p, is increased in HCC samples (*n* = 54) [20] Surely, the role of miRNA is multivariate and it may induce gene expression in some cases, but for today these cases are insufficiently studied [21]. Common target genes for two others mostly changed miRNAs – decreased miRNA-122-5p and increased miRNA-221-3p - are estrogen receptor 1 (ER-1) and forkhead box P1 (FOXP1). The expression level of both of these target genes - *ER-1* and *FOXP1* - may be increased in HCV-related HCC samples compare to normal tissue [22, 23]. Anyway, gene expression is a very complex process and miRNAs are not the main factor in gene expression, along with the fact, that the expression levels data of the miRNAs and their target genes are controversial make us considering adding other parameters for including a miRNA to the marker panel. However, increasing in expression levels of miRNA-21-5p and miRNA-221-3p were also found in other studies of liver cancer samples. [24–26].

At last, remembering the primary liver cancer has a strong dependance on geography, its incidence rate is relatively low in Russian Federation [3]. This relative low incidence rate along with other factors including unwillingness of patients of the risk group to come systematically to the research center for blood collection without personal interests make the prospective cases database formation an issue in the Russian Federation. Considering the fact, the Liver Research Center of the RUDN Medical Institute has a database of patients with HCV-associated cirrhosis with the contact information, a possible solution to this problem could be the use of a biomaterial that does not require the presence of medical personnel for its collection, for example, such as a saliva. In our research, we proved saliva was a promising approach as a source for exosomal and non-exosomal miRNAs. The exosomal miRNAs normalized to exosomal miRNA-16-5p seem to be the form of miRNA normalization, which give the strongest association with primary liver cancer in saliva samples.

## MATERIALS AND METHODS

This is a case-control study performed in the Liver Research Center of Institute of Medicine of Peoples’ Friendship University of Russia (RUDN). Patients were informed of the purpose of the acquisition of anonymized clinical data, obtainment of blood and saliva samples and future publication of collected data prior to enrolment in the research project. Informed consent was obtained from all patients. The study was approved by ethics committee of Institute of Medicine of RUDN.

The control group consisted of 21 volunteers: age 30–60, median 46,0 ± 9 (12 male: age 32–60, median 47,5 ± 8,4; 9 female: age 30–59, median 44 ± 9,3), meeting the following criteria: no hospitalization during last 12 months, no cancer/autoimmune disease/alcoholism/drug addiction in anamnesis, no pregnancy or lactation. The first study group (hereinafter, cirrhotic group) consisted of 24 patients with HCV-related liver cirrhosis: age 35–55, median 45,5 ± 6,8 (15 male: 35–55, median 44 ± 7,4; 9 female: 35–53, median 46 ± 6,3) and the second study group (hereinafter, cancer group) consisted of 24 patients with liver cancer: age 43–65, median 49,5 (Q1:44, Q3:58,2); (14 male: age 43–62, median 49,0 (Q1:44, Q3:57); 10 female: age 44–65, median 52,0 ± 8,6). Liver cirrhosis was diagnosed by transient elastography method (METAVIR F4, FibroScan), HCV status was positive in anamnesis, all patients with HCV-related cirrhosis were treated with direct acting antivirals and are HCV negative during more than 1 year, liver cancer was diagnosed by contrast-enhanced MRI with liver-specific contrast agents, 15 cases were identified as hepatocellular carcinoma by pathomorphological examination, 9 cases missed the results of pathomorphological examination at the time of this writing.

Peripheral venous blood was collected in EDTA K2 4 ml tubes from volunteers, patients with liver cirrhosis and liver cancer, immediately after collection blood samples were centrifugated at 3000 g for 10 minutes at room temperature (Eppendorf 5810R, rotor A-4-81, Germany), two aliquots for each patient were frozen at –80°C. Before saliva obtainment patients undergo 2 hours fasting, saliva was obtained in 50 ml tube, about 1-2 ml was transferred to 1.5 ml tubes and centrifugated at 3000 g for 10 minutes at room temperature (Eppendorf Minispin^®^, Germany), 2 aliquots of supernatant of each sample were collected and frozen at –80°C.

Further exosomal and non-exosomal miRNAs were isolated both from plasma and saliva samples. In order to obtain these miRNA fractions, exosomes were isolated from one aliquot of every sample and proceeded for miRNA isolation, another aliquot of the same sample was ultracentrifugated and exosome-depleted supernatant proceeded for miRNA isolation. Hereinafter, miRNAs, that are isolated from the exosomes are named exosomal miRNA; miRNAs, that are isolated from the exosome-depleted supernatant samples are named non-exosomal miRNAs.

In order to obtain exosomal miRNA fraction exosomes from plasma and saliva samples were isolated with miRCURY Exosome Serum/Plasma Kit (Qiagen, Germany). After thawing saliva and plasma samples were centrifugated at 8000 g for 10 minutes and at 10 000 g for 30 minutes, in order to enrich for microvesicles both saliva and plasma samples (400 μL) after centrifugation were passed through a 0.22 μm filter [27]. The resulting filtrates were processed to exosome isolation, in brief, filtrates were supplemented with PBS till 0.5 mL. Further isolation process was performed according to the manufacturer’s protocols finishing with samples incubation at 4°C for 1 h and centrifugation at 500 g for 5 min (protocol plasma) at room temperature. Pellet fraction was resuspended in 270 μL of Resuspension buffer and frozen at −80°C. These enriched for exosomes samples were named exosomal samples. In order to obtain non-exosomal miRNA fraction, aliquots of plasma and saliva samples after thawing were centrifugated at following regimen: 300 g 10 minutes, 2000 g 10 minutes, 10,000 g 30 minutes, 150,000 g 70 minutes, 150,000 g 70 minutes, at every step the supernatant was taken (Optima Max E, MLA-150 rotor, Beckman Coulter, Inc., CA, USA) [28]. These exosome-depleted samples were proceeded to following miRNA isolation.

8 random exosomal samples and 8 random exosome depleted samples of every group were analyzed with photon cross-correlation spectroscopy (NANOPHOX, Sympatech GmbH, Germany) in order to identify the size spectrum of isolated vesicles. All exosomal and exosome depleted samples were examined with CD63 based ELISA (ExoELISA-ULTRA Complete Kit, System Biosciences, CA, USA) (Table 1).

MiRNAs were isolated with similar kit from both exosomal and exosome-depleted plasma and saliva samples according to the manufacturer’s instruction (miRNeasy Serum/Plasma Advanced kit, Qiagen, Germany), miRNA concentration in samples after isolation was estimated by ultra-violet visible spectrophotometry in “microRNA” mode (NanoPhotometer^®^ NP80, Implen GmbH, Germany). Reverse transcription reactions were performed using a SYBR miRNA Reverse Transcription kit (miRCURY LNA™ RT Kit, Qiagen, Germany). qPCR was performed using CFX96™ Real Time System (Bio-Rad, CA, USA). Each reaction mixture for qPCR contained 5 μL of master mix, 1 μL miRNA-specific LNA primer and 4 μL diluted RT product (1:80) in a total volume of 10 μL. For both plasma exosomal and non-exosomal depleted samples LNA primers for following miRNAs were used: let-7a-5p, miR-16-5p, miR-18a-5p, miR-21-5p, miR-22-3p, miR-34a-5p, miR-103a-3p, miR-122-5p, miR-145-5p, miR-221-3p, miR-222-3p, miR-224-5p. For both saliva exosomal and exosome-depleted samples LNA primers for following miRNAs were used: let-7a, miRNA-21-5p, miRNA-122-5p. Reactions were carried out using the following thermal cycling parameters: 95°C for 10 min, followed by 40 cycles of 95°C for 10 s, and 60°C for 1 min, followed by holding at 4°C. Raw data were analyzed using Bio-Rad CFX Manager Software version 3.1 (Bio-Rad, CA, USA), generally using the automatic Ct setting for assigning baseline and threshold values for Ct determination. Those miRNAs that appeared on the profile with *C*_t_ value of < 35 were considered as ‘detected’. The expression level of each miRNA was normalized to miRNA-16 expression level, exosomal and non-exosomal miRNA ratio was calculated.

## CONCLUSIONS

In this study we have realized that non-exosomal miRNAs normalized to non-exosomal miRNA-16p in plasma samples are stronger associated with primary liver cancer, than exosomal miRNAs. Three miRNAs with the mostly pronounced change of expression levels in plasma in liver cancer samples – miRNA-21-5p, 122-5p, 221-3p – were detected in saliva. In contrast to plasma samples, in saliva exosomal miRNA normalized to exosomal miRNA-16-5p showed stronger association with liver cancer, than non-exosomal miRNAs. Thus, saliva may be a promising approach for collection of prospective primary liver cases for the search of prognostic miRNAs.
